# Classification of Heart Sounds Using Chaogram Transform and Deep Convolutional Neural Network Transfer Learning

**DOI:** 10.3390/s22249569

**Published:** 2022-12-07

**Authors:** Ali Harimi, Yahya Majd, Abdorreza Alavi Gharahbagh, Vahid Hajihashemi, Zeynab Esmaileyan, José J. M. Machado, João Manuel R. S. Tavares

**Affiliations:** 1 Department of Electrical Engineering, Shahrood Branch, Islamic Azad University, Shahrood 43189-36199, Iran; 2 School of Surveying and Built Environment, Toowoomba Campus, University of Southern Queensland (USQ), Darling Heights, QLD 4350, Australia; 3 Faculdade de Engenharia, Universidade do Porto, Rua Dr. Roberto Frias, s/n, 4200-465 Porto, Portugal; 4 Departamento de Engenharia Mecânica, Faculdade de Engenharia, Universidade do Porto, Rua Dr. Roberto Frias, s/n, 4200-465 Porto, Portugal

**Keywords:** biomedical signal, phonocardiogram, deep learning, signal to image transform

## Abstract

Heart sounds convey important information regarding potential heart diseases. Currently, heart sound classification attracts many researchers from the fields of telemedicine, digital signal processing, and machine learning—among others—mainly to identify cardiac pathology as quickly as possible. This article proposes chaogram as a new transform to convert heart sound signals to colour images. In the proposed approach, the output image is, therefore, the projection of the reconstructed phase space representation of the phonocardiogram (PCG) signal on three coordinate planes. This has two major benefits: (1) it makes possible to apply deep convolutional neural networks to heart sounds and (2) it is also possible to employ a transfer learning scheme by converting a heart sound signal to an image. The performance of the proposed approach was verified on the PhysioNet dataset. Due to the imbalanced data on this dataset, it is common to assess the results quality using the average of sensitivity and specificity, which is known as score, instead of accuracy. In this study, the best results were achieved using the InceptionV3 model, which achieved a score of 88.06%.

## 1. Introduction

Recent studies show that cardiovascular diseases (CVDs), with approximately 18 million deaths annually, have become one of the leading causes of mortality. Thus, the early detection of heart disease is an important issue due to its crucial role in saving people’s lives. This puts studies in related fields as a canonical problem involving various fields of research and applications.

Various CVDs and cardiac pathologies, such as coronary heart disease, congenital heart disease, and peripheral arterial disease, can be diagnosed through cardiac auscultation. Additionally, most heart diseases related to heart valves are detectable through the phonocardiography (PCG) signal. Therefore, the automatic detection of cardiac abnormalities through PCG signals has attracted the interest of many researchers. It can be considered a multidisciplinary research topic involving telemedicine; digital signals, i.e., audio processing; and machine learning. Hence, this research field aims to analyse the PCG signal, i.e., the heart sound, to detect any abnormality in heart functioning. This is known as PCG signal classification, mainly into normal or abnormal signals.

**Figure 1:**
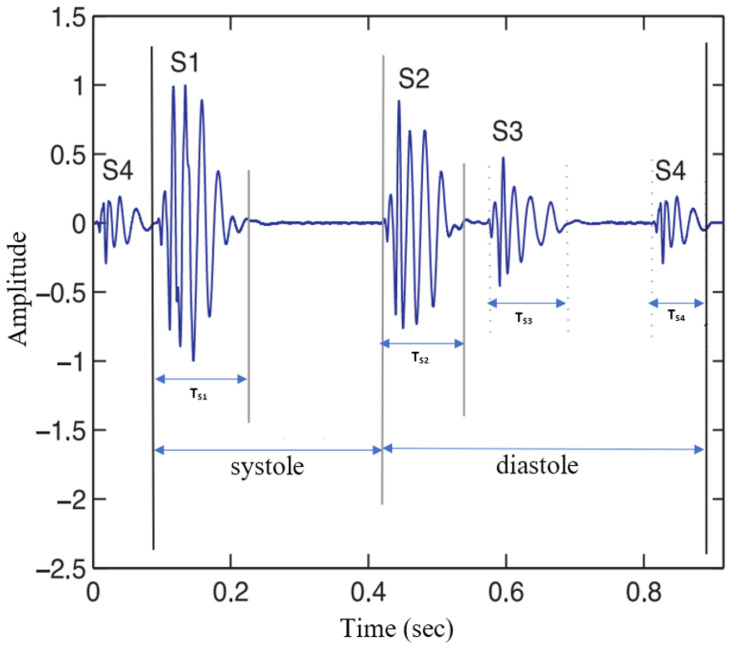
Typical waveform of a phonocardiogram (PCG) signal and its components: S1 ($T_{S1}$ = 70–150 ms), S2 ($T_{S2}$ = 60–120 ms), S3 ($T_{S3}$ = 40–100 ms), and S4 ($T_{S4}$ = 40–80 ms).

Each period of the PCG signal comprises two major states: systole and diastole. Systole, with a typical time duration of 300 ms, consists of a 70–150 ms S1 state, wherein the mitral and tricuspid valves are closely followed by a silent segment. Diastole, with a typical duration of 500 ms, includes the S2 state related to the closure of aortic and pulmonary valves and high-frequency S3 and S4 noises that are usually not heard. Figure 1 illustrates a typical PCG signal and its four states: S1, S2, S3, and S4.

Figure 1 shows how different heart symptoms affect the temporal dynamics of PCG signals. Accordingly, while heart sound segmentation algorithms aim to segment the four major states of the PCG signal, heart sound classification algorithms tend to develop machine learning-based algorithms to classify normal heart sounds versus abnormal heart sounds. It is common to detect abnormal heart function through electrocardiogram (ECG) signal analysis. However, acquiring PCG signals is more straightforward, cheaper, and less invasive than ECG signals. Additionally, analysing PCG signals allows experts to check the patient and help to make a quick initial decision about the patient’s condition.

There are three main tasks related to the processing and analysis of PCG signals: signal denoising, segmentation, and classification. Denoising aims to provide a clean PCG signal using, for example, noise cancellation techniques. It is therefore common to use denoising as a preprocessing stage. Segmentation detects the four major states of the PCG signal throughout each cardiac cycle, and, finally, PCG classification categorises the heart functioning under analysis into normal and abnormal.

According to the classic point of view of the PCG or similar audio classification problems, the current problem can be addressed based on two major steps: finding specifications, i.e., feature extraction, and their categorisation, i.e., classification. Thus, first, in the preprocessing step, a noise cancellation method is usually applied to remove noise [1,2], and then, the temporal–spectral information is gathered as numerical data. The extraction of discriminative features is one of the main challenges in this area. Mell Frequency Spectrum Coefficients [3,4,5,6], Hilbert–Huang transform [7], S transform [8], Wavelet transform [9,10,11,12], and multi-domain features [13] are well-known feature extractors that have been proposed for PCG classification.

Usually, the parameters of the used classification model are adjusted to classify normal versus abnormal signals during its training phase. Then, in the test phase, the trained model is employed to predict whether an input signal is normal or abnormal. In contrast to the classic methods, deep learning algorithms have partially solved the feature extraction issue by implementing networks that can automatically derive features from the raw input signal throughout their pipeline. Hence, these kinds of algorithms learn to extract discriminative features through training; their only disadvantage is the requirement of large-scale training datasets and proper hardware. A comprehensive survey on employing deep neuronal networks (DNNs) for PCG signal classification is given, for example, in [14].

Many related works use the raw signal as the network’s input [15]. On the other hand, others perform a feature extraction task before inputting the signal to the network. For example, in [5], low-level features were extracted from the input PCG signal, and then a DNN was applied to derive high-level features and learn their relationship with the output classes. Furthermore, various DNN models and architectures have been proposed for this task. In [5], a long-short term memory (LSTM) DNNs was employed. In [5], recurrent neural networks (RNNs) were used, while [16] suggested autoencoders for the classification of heart sounds. On the other hand, although some researchers use one-dimensional (1D) convolutional neural networks [17,18], others suggest deep convolutional neural networks (DCNNs). While DCNNs are the most favourable networks for image processing, some researchers try to apply them to other signal processing problems. To this end, converting the original one-dimensional signal into corresponding two-dimensional (2D) tensors is recommended before applying the DCNN to benefit from the advantages of 2D convolutions [19]. In [20], frequency features were extracted using a CNN, and temporal features were extracted using a recurrent neural network. Then, the fusion of these features was inputted into a lightweight neural network for heart sound classification. In [21,22,23], the authors suggested time-frequency representation of the original signal such as a spectrogram image as a proper input for 2D DCNNs. For example, in [24], a signal-to-cycle conversion preprocessing step was employed to build the spectrogram based on such signal cycles instead of fixed-length time frames. Then, a hybrid classifier composed of AlexNet and SVM was employed, which achieved promising results even using just 2–3 s of data. Regarding the success of DCNNs in various applications, the motivation for this study is the effective application of DCNNs for classifying PCG signals. Therefore, one should adopt the PCG signal as a proper input for DCNNs.

Despite the success of DCNNs in various applications, these models comprise a large number of trainable parameters which demand a high amount of memory and processing capacity. Some studies suggest the use of network pruning and weight quantization to address this issue [25,26], which allows for the implementation of DCNNs on simple processors such as microcontrollers. However, in this study, the main focus was more on the classification performance and not on the computational complexity, which will be addressed in the near future.

Based on our knowledge, previous studies mostly convert the original one-dimensional signal into a 2D spectrogram image using techniques based on a fusion step. Therefore, the main contribution of this study is the representation of the original one-dimensional PCG signal as a 2D chaogram image and input this image into a pretrained DCNN model. Chaogram was first introduced in [27] for speech emotion recognition. It uses a reconstructed phase space, a well-known nonlinear dynamic processing tool useful in analysing chaotic systems, to convert the input PCG signal into a 2D image. Therefore, the chaogram transform can reflect the chaotic behaviour of a non-linear dynamic system on the output RGB image. Moreover, it allows for using 2D DCNNs to classify PCG signals.

The remainder of this article is organised as follows. The details of the proposed approach are given in Section 2. To assess the effectiveness and efficiency of the proposed approach, a group of experiments was designed and performed, and the experimental setup and results are presented in Section 3. Finally, the conclusion is provided in Section 4.

## 2. Proposed Approach

**Figure 2:**
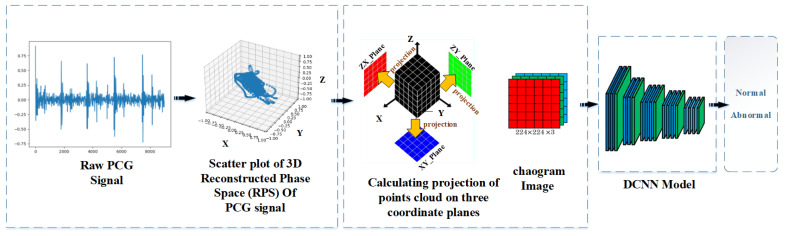
Flow diagram of the proposed approach for PCG signal classification: (1) building RPS from the original PCG signal; (2) projections of data represented in RPS are calculated on the XY, XZ, and YZ coordinate planes to form the red, green, and blue channels of the corresponding RGB image; (3) the chaogram is formed as a compatible input for a deep convolutional neural network; (4) a DCNN model pretrained on the large ImageNet Dataset is used for learning the extraction and classification of the chaogram’s high-level features; (5) the PCG signals are classified as normal or abnormal signals using the trained DCNN model.

The proposed approach for computer-aided heart auscultation comprises a chaogram transformation applied to the original PCG signal and a DNN model that learns features and classifies them. Figure 2 depicts the proposed approach, which is described in detail as follows: After describing the two-folded preprocessing scheme in Section 2.1, Section 2.2 describes the transformation of the original one-dimensional PCG signal into the corresponding 2D chaogram image; finally, the pretrained convolutional neural network (CNN) model that was used is described in Section 2.3.

### 2.1. Preprocessing

The heart sound is affected by other sounds of the human body. Therefore, it is necessary to employ preprocessing and noise cancellation techniques. Whitaker et al. used a two-stage noise cancellation technique. The authors used a third-order Butterworth band-pass filter with bandwidths of 15 to 800 Hz. Then, the spectral subtraction denoising scheme was employed [28]. The authors used adaptive filters based on noise power outside the expected range of the heart sound spectrum, which performed well in removing background noise presented in biological signals such as speech and EEG. Finally, the clean PCG signal was obtained by subtracting the weighted version of predicted noise power from the PCG spectrum [28].

### 2.2. Transformation of One-Dimensional PCG Signal into a Chaogram Image

**Figure 3:**
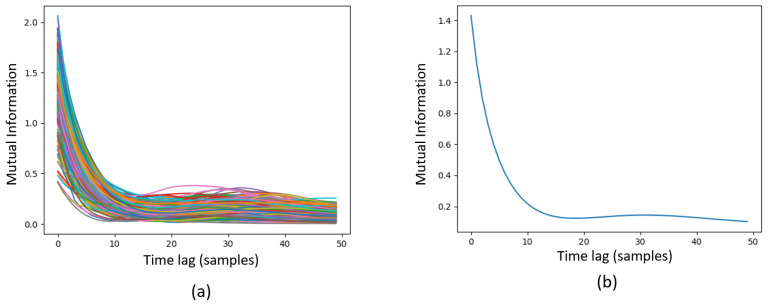
Mutual information of all samples in the PhysioNet dataset (**a**) and their average mutual information (**b**).

To build a chaogram image, reconstructed phase space (RPS) is a beneficial tool for analysing a system’s nonlinear and chaotic behaviour [29]. A phase space comprises the collection of all possible states of a system. However, determining all the possible states of real systems is usually impossible. Instead, the output signal of the system under study can be used to reconstruct its phase space. This signal inherits the main characteristics of its system. Here, the PCG signal is considered to be the output of the heart, i.e., the main system; then, the RPS can be built by defining the vectors:

(1) $$\bar{S_{n}}=\left[S_{n},S_{n+\tau },S_{n+2\tau },\ldots ,S_{n+(d-1)\tau }\right],$$

where $S_{n}$, with n=1,2,3…N, is the *n*th sample of the PCG signal; *d* and τ denote the embedding dimension and time delay, respectively; and $\bar{S_{n}}$ is a linear vector that specifies one of the possible states of the system as a point in RPS, which is determined by the consecutive collection of such vectors as:

(2) $$S={\left[\bar{S_{1}},\bar{S_{2}},\ldots ,\bar{S_{n}}\right]}^{T},$$

where *S* denotes the RPS, and *T* stands for the transpose operation. One of the central issues in RPS is determining the optimal values for τ and *d* based on mutual information and the false nearest neighbour algorithm, respectively [29]. The mutual information between two *X* and *Y* signals can be calculated as:

(3) $$I\left(X,Y\right)=\sum_{y\in Y}\sum_{x\in X}P_{\left(X,Y\right)}\left(x,y\right)\log\left(\frac{P_{\left(X,Y\right)}\left(x,y\right)}{P_{\left(X\right)}\left(x\right)P_{\left(Y\right)}\left(y\right)}\right),$$

where p(x) stands for the probability density function of *x*, and p(x,y) denotes the joint probability density function of *x* and *y* variables. Here, for all the samples in the PhysioNet dataset, the mutual information of the PCG signals and their corresponding delayed versions were calculated for the time lags of 0 (zero) to 50, as shown in Figure 3. As seen from the graphs in Figure 3, the first minimum of mutual information offered by different samples is a digit between 10 and 20. According to Figure 3, the first minimum of mutual information in the averaged curve is placed on the time lag of 18. Therefore, it was considered that τ=18. Although, based on the experiments performed in this study, the optimum dimensionality for embedding dimension for PCG signals is 4, we considered that d=3 in order to build the used chaogram images.

**Figure 4:**
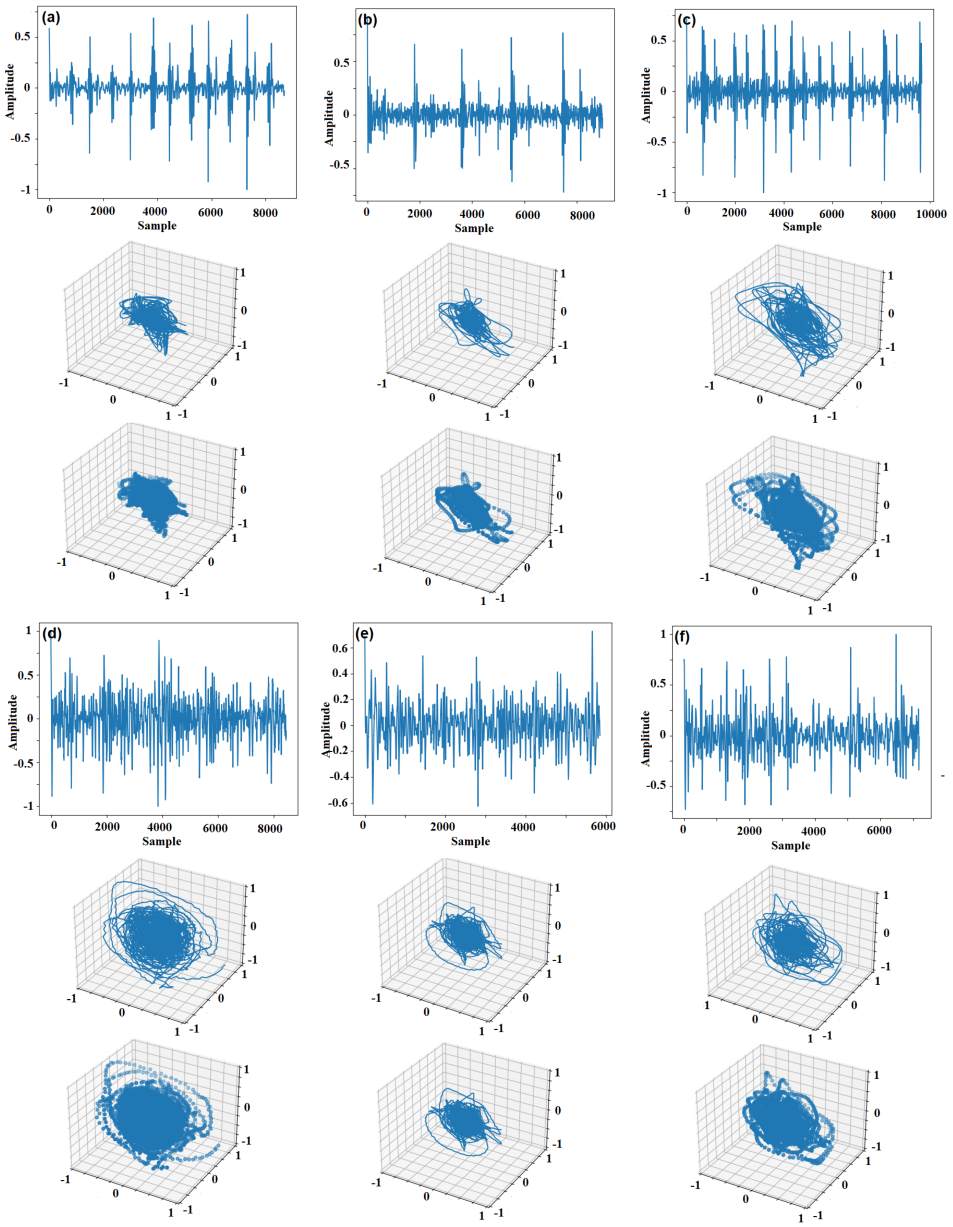
Six PCG signals and their representation in RPS with τ=18 and d=3: (**a**–**c**) are normal, and (**d**–**f**) are abnormal signals.

The RPS of a PCG signal is considered to model the nonlinear dynamics of the heart as the system that generates the PCG signal. Observations also confirm that the patterns formed in the RPS of PCG are strongly correlated with the heart’s functioning condition. Figure 4 shows six PCG signals (three normal and three abnormal) and their representation in RPS (with τ=18 and d=3). In the original RPS, a curve goes from one point to another according to a sequence (rows 2 and 5 of Figure 4). By eliminating the links between the points, the RPS would be converted into a set of points in a 3D space (rows 3 and 6 of Figure 4). The shapes of these data points form cloud-shape patterns giving fundamental information about the corresponding system [30].

**Figure 5:**
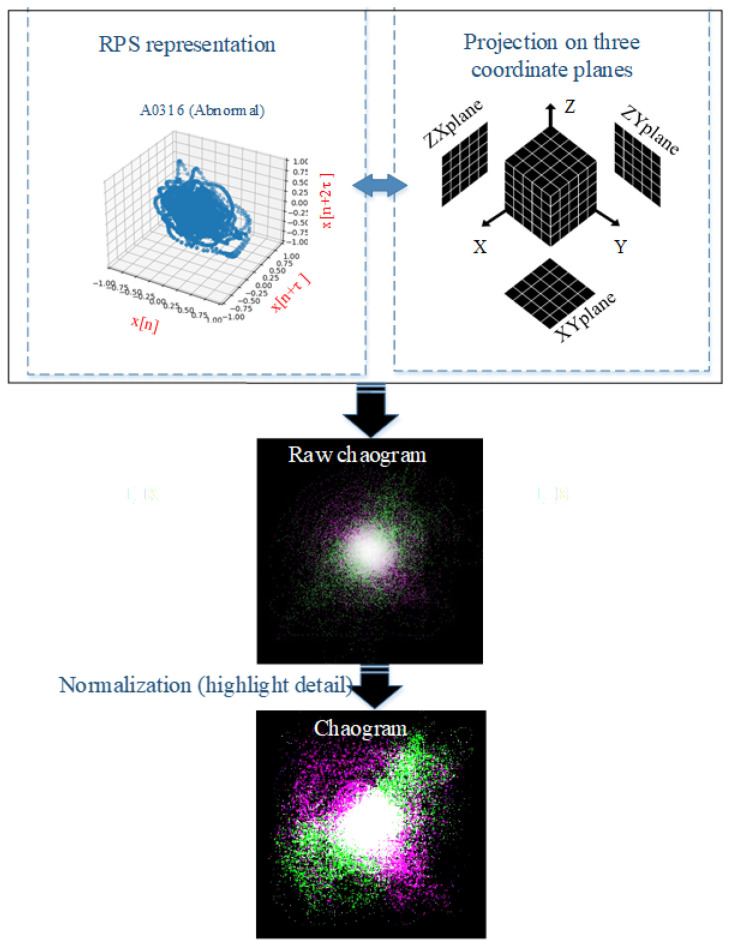
Extracting the chaogram image from the reconstructed phase space of a PCG signal.

When the RPS of a PCG signal is calculated using Equation (2), the space is partitioned into parts in each direction. Consequently, the space is a 224×224×224− cell net, while each vector in Equation (1) is considered a point in this net. Then, the frequency of points inside each cell is calculated to form a 3D tensor, *T*. After that, the projections of this 3D tensor on XY,XZ, and YZ planes are calculated as three images, Ixy,Ixz, and Iyz, as:

(4) $${I_{x}}_{y}\left(i,j\right)=\sum_{K=1}^{224}T(i,j,k),$$

(5) $${I_{x}}_{z}\left(i,k\right)=\sum_{j=1}^{224}T(i,j,k),$$

(6) $${I_{y}}_{z}\left(j,k\right)=\sum_{i=1}^{224}T(i,j,k),$$

where *T* denotes the 3D tensor. Consequently, three images with the size of 224×224 are obtained. Finally, these three images act as colour channels of an RGB image to build the chaogram image. An image enhancement stage is employed to emphasise the weak details in these images. Figure 5 shows the flow diagram of this process.

**Figure 6:**
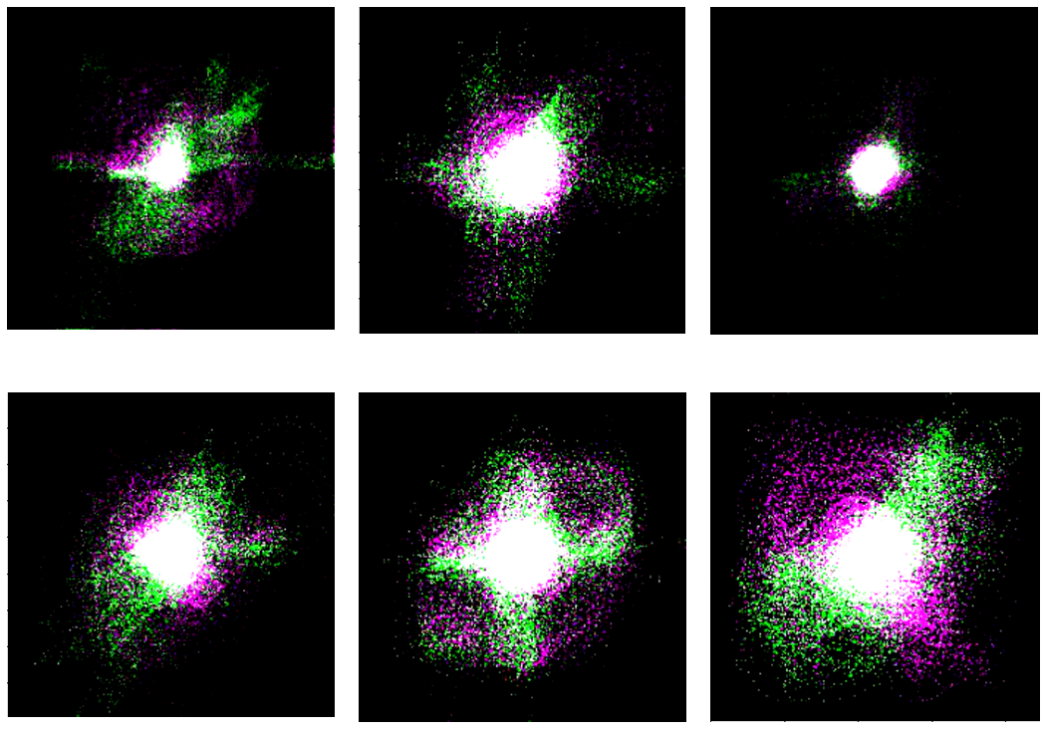
Chaograms of six PCG signals: on the top row, 3 normal cases, and, on the bottom row, 3 abnormal cases.

This study employs a DCNN to learn the relationship between the cloud shapes and patterns in the chaogram image and the heart functioning condition. Since the size of the PhysioNet dataset is relatively small, the training of a deep neural network with a considerable number of variables is impossible since the risk of overfitting is unavoidable. Therefore, pretrained networks are recommended based on a transfer learning (TL) scheme. Hence, the chaogram images had to be of the same size as the input data admitted by the employed model. Hence, the size of the chaogram images must be equal to the input size admitted by the used VGG DCNN model, which is a 224×224×3  RGB image, if it is intended to employ it as a pretrained model. Figure 6 shows the chaogram images of the 6 PCG samples shown in Figure 4.

As shown in Figure 6, while the patterns of the chaogram images shown in each row resemble each other, the images of the two rows differ. In other words, it seems that the chaogram images provide a high intraclass similarity and interclass difference.

### 2.3. DCNN Model

In this study, four pretrained deep learning models, AlexNet, VGG16, InceptionV3, and ResNet50, were evaluated for the classification of PCG signals using chaogram images. The AlexNetDCNN model [31] won the ImageNet ILSVRC−2012 challenge. It comprises five convolutional and three fully connected layers. The VGG16DCNN model gained the second rank in the ImageNet ILSVRC−2014 challenge with a slight difference from the first model: GoogLeNet network [32]. VGG16 includes 16 convolutional layers. GoogLeNet consists of nine Inception modules with trainable hyperparameters. Inceptionv3, the upgraded version of Inception, has 11 Inception modules [33]. On the other hand, ResNet50 (residual network) consists of 50 layers [34], and it won the ImageNetILSVRC−2015 challenge.

As already mentioned, training the four deep learning networks from scratch on the relatively small sample size dataset, PhysioNet, increases the risk of overfitting. Therefore, to reduce this risk, the transfer learning technique—where only the two last layers of the network were allowed to fine-tune on the used dataset, while the earlier layers were kept unchanged with the weights pretrained on the large-scale ImageNet dataset—was employed. Transfer learning is a common technique used in deep learning, particularly in DCNN models, since it can speed up the learning rate and improve the model’s generalisation [35,36]. Additionally, data augmentation was used in this study to enrich the used training dataset by building new samples by applying various transformations and deformations to the original samples. Therefore, the rotation of the chaogram image (with angles of 5, 10, 15, 20, 25, and 30 degrees), scale (with factors of 1.05, 1.1, 1.15, and 1.2), width shift (by 5, 10, 15, 20, and 25 pixels), and height shift (by 5, 10, 15, 20, and 25 pixels) were applied to the original images to generate the final training data. As a result, 20 (6×4×5×5) new versions for each original sample were generated, and, in total, the number of the training samples increased 21 times: from 2868 to 60,228 (2868×21) samples. Additionally, to also reduce the risk of overfitting, the dropout technique was used, where each neuron is selected to be removed with a predefined probability. It makes the learning process more independent from the neurons and simplifies the network, thus preventing overfitting. A dropout layer with p=0.5 was used here to implement this technique.

The fine−tuning process in a DCNN network is controlled with a set of hyperparameters, including, optimiser functions: Adam, SGD, RMSprop, Adadelta, Adagrad, Adamax, Nadam, and Ftrl; learning rate: $1\times 10^{-5}$ to $1\times 10^{-1}$ according to steps of $1\times 10^{-5}$; batch size: 50 to 500 according to steps of 2; and epochs: 50 to 400 according to steps of 20. These parameters can be selected based on a trial-and-error method. However, optimising such hyperparameters can improve the classification model’s performance. In this study, the Bayesian optimisation algorithm [37] in the “$bayes_{o}pt$” package in Python was used for the fine−tuning of the hyperparameters. The stopping condition for the optimisation algorithm was when there was no improvement in 20 consecutive epochs.

## 3. Experimental Setup and Results

### 3.1. Experimental Setup

The proposed approach was evaluated on the PhysioNet dataset. A computational system with an AMDRYZEN9Corei9−4900HCPU, a NVIDIA−GTX1660Tigraphicscard, and 16GB DDR4 of RAM was used. All simulations were performed in the Anaconda environment using SpyderIDE, and the algorithms were coded in Python. The phase space reconstruction was implemented using the skedm library. In addition, pretrained DCNN models were implemented using the TensorFlow framework and Keras library [38].

### 3.2. Dataset

The PhysioNet/CinC [39] dataset was used to evaluate the proposed approach. The included samples are not all similar due to different recording environments, equipment, and time duration. The dataset consists of 2868 PCG signals, including 2249 normal and 619 abnormal samples that were resampled to 2000 Hz and saved in ".wav” format [39]. The samples are organized into five subfolders (a, b, c, d, and e), with significant differences and variations in each subfolder due to their origin and recording condition. This dataset was built aiming the classification of heart sounds, such as in this study and in many other related works that also used it [5,10,40].

### 3.3. Evaluation Metrics

To evaluate the performance of the classification process, the confusion matrix was computed with the abnormal cases as the positive class, i.e., the normal cases, and the sensitivity, specificity, and accuracy were calculated using:

(7) $$Sensitivity\;\left(Se\right)=\frac{TP}{TP+FN},$$

(8) $$Specificity\;\left(Sp\right)=\frac{TN}{TN+FP},$$

(9) $$Accuracy=\frac{TP+TN}{TP+TN+FP+FN}.$$

where TP,TN,FP, and FN are the confusion matrix components representing true positive, true negative, false positive, and false negative cases, respectively. Since the data in the PhysioNet dataset is imbalanced, the accuracy tends to be biased toward the majority class and cannot measure misclassification costs. Hence, it is not the most suitable measure to be used in the evaluation of the classification performance. Therefore, the average between sensitivity and specificity was chosen as an alternative official evaluation metric for the 2016 PhysioNet/Computing in Cardiology Challenge, which is calculated as:

(10) $$Score=\frac{Se+Sp}{TP+TN+FP+FN}.$$

### 3.4. Experimental Results

The experiments were performed using the hyperparameters selected by the Bayesian optimisation algorithm, as reported in Table 1. Table 2 presents the results of the experiments, which were performed based on the 5-fold cross-validation technique.

According to Table 2, all tested models performed well in classifying the used PCG signals using the built chaogram images. This indicates the ability of the proposed transformation to convert PCG signals to RGB images. In other words, it implicitly shows that the employed models can efficiently extract useful features from the built chaogram images and consequently obtain results with a high classification rate. Among the employed models, the InceptionV3 model obtained the highest score of for accuracy (88.06), sensitivity (84.49), and specificity (91.63). On the other hand, the lowest accuracy rate was obtained by AlexNet.

However, although it seems pretty logical, what was the cause of the difference between the results of the four models? Undoubtedly, one can think that the main reason can be the different architectures of the used models. The optimum depth of a network model depends on the size of the used dataset or the number of trainable parameters, while a few trainable parameters of a too-shallow network may not be enough for learning the general rules of a complicated classification problem. In a deep network with numerous trainable parameters, the models may learn examples instead of rules, which is called overfitting. Therefore, depending on the size of the used dataset and complexity of the problem, the appropriate network model should be carefully selected.

To compare the proposed method against state-of-the-art methods, it was implemented according to same conditions of the ones suggested in [41,42,43,44]. Table 3 compares the results obtained by the proposed method and the ones obtained by state-of-the-art methods. Based on the data presented in Table 3, one can realize that the proposed method shows better accuracy and recall than all other methods. The precision of the proposed method is slightly lower than the ones of the other methods, and the f1 score is only lower than the one of [44]. The best recall shows that the proposed method led to the lowest false positive rate, which is the most important parameter in medical care. In future works, adding a postprocessing or fusion step to the output is recommended to increase the precision and F1 score of the proposed method.

## 4. Conclusions

In this article, a new approach for the classification of PCG signals was proposed; its main contribution is the use of 2D chaogram images to detect abnormalities from heart sounds. From the experiments conducted, it is possible to use a vast range of image processing tools to analyse one-dimensional signals. For instance, this study employed two-dimensional DCNNs to analyse PCG signals from the corresponding built 2D chaogram images. Second, the chaogram images derived from the reconstructed phase space of the PCG signal under analysis inherited the main topological features of the cloud-shaped patterns in that space. Hence, they tend to carry important information about the nonlinear chaotic behaviour of the PCG signal. According to the observations made during this study, the built chaogram images are similar in each normal and abnormal class, while they differ from one class to another. In other words, this provides high intraclass similarity and interclass distance. Consequently, the convolutional masks in DCNN layers can extract meaningful information from those images. Third, converting PCG signals to chaogram images reduces the risk of overfitting due to the possibility of using transfer learning and pretrained DCNNs. This reduces the trainable parameters while effectively maintaining the classification model’s complexity. Therefore, complex problems with a few training samples can be solved with less risk of overfitting.

Furthermore, due to the imbalanced distribution of the data included in the used PhysioNet dataset, the accuracy cannot correctly reflect the classification performance. Instead, the score factor, which is determined as the average of sensitivity and specificity, was used to evaluate the classification performance. According to the experiments conducted, the use of different pretrained DCNNs did not greatly impact the achieved score factor. The best score was achieved using InceptionV3, and the lowest score was obtained by AlexNet: 88.06% and 86.88%, respectively. The small difference between these values confirms the suitability of the chaogram transform to represent the used heart sound data. On the other hand, the structure of the used model had a considerable impact on the final results of the proposed method. This is related to both operators employed in each layer of the used classifier model, the depth of its layers, and the corresponding number of trainable parameters. If the number of trainable parameters is too large, the model tends to be overfitted to the training data, which can be avoided if transfer learning is employed to pretrain the used model.

To build the corresponding chaogram image, the PCG signal is represented in the 3D reconstructed phase space. However, it is recommended to determine the optimum embedding dimension by using a false nearest algorithm, which is one of the main limitations of the current method. Hence, a potential future study could investigate a solution that can build the chaogram images based on the raw signal’s optimal dimensional reconstructed phase space as an effective step in generalizing this paper’s proposed method. For example, dimensional reduction approaches such as principal component analysis can be beneficial in this regard. Moreover, none of the employed DCNNs were optimized for classifying PCG signals. Thus, future works can endeavour to design a new dedicated neural network architecture that can increase classification accuracy.

## Figures and Tables

**Table 1:** Results of the hyperparameter optimisation.

Hyperparameter	AlexNet	VGG16	InceptionV3	ResNet50
Optimizer function	Adam	RMSprop	Adamax	Adam
Learning Rate	$10\times 10^{-5}$	$10\times 10^{-3}$	$10\times 10^{-5}$	$10\times 10^{-4}$
Batch size	98	64	102	86
Epoch	260	320	280	260

**Table 2:** Results regarding the classification of the PCG signals included in the PhysioNet dataset (best found values are in bold).

Network	Sensitivity	Specificity	Accuracy	Score
AlexNet	82.55	91.21	89.68	86.88
VGG16	83.36	91.49	90.05	87.43
InceptionV3	**84.49**	**91.63**	**90.36**	**88.06**
ResNet50	83.68	91.39	90.02	87.54

**Table 3:** Comparison among the results obtained by the proposed method and those obtained by state-of-art methods.

Algorithm	Accuracy	Recall	Precision	F1 Score
[41]	77.38	-	-	-
[42]	-	86.69	92.77	-
[43]	-	90.32	91.82	81.5
[44]	91.32	89.52	94.37	90.12
Proposed	92.19	90.95	90.95	83.41

## Data Availability

Not applicable.
